# Reference percentiles for evaluating phase angle using bioelectrical impedance analysis in Chinese children aged 6–11 years

**DOI:** 10.3389/fnut.2025.1597087

**Published:** 2025-07-31

**Authors:** Jiongxian Yang, Yue Qian, Xiping Gu, Yue Li, Chenlu Yang, Aimin Liang

**Affiliations:** ^1^Department of Health Care Center, Beijing Children’s Hospital, Capital Medical University, National Center for Children’s Health, Beijing, China; ^2^Department of Pediatrics, People’s Hospital of Tianzhu Tibetan Autonomous Country, Wuwei City, Gansu Province, China

**Keywords:** bioelectrical impedance analysis (BIA), phase angle (PhA), children, Chinese, reference centile curves

## Abstract

**Background:**

Bioelectrical impedance analysis (BIA)-derived phase angle (PhA) is influenced by multiple factors including age, sex, ethnicity, and growth status. However, reference ranges for PhA in Chinese children remain undefined. This study was designed to establish normative PhA values in Chinese school-aged children, with the goal of providing clinicians with more accurate nutrition assessments.

**Methods:**

A retrospective analysis was performed. Primary diagnosis reports were gathered from the hospital information system (HIS). The data were stratified by age and sex, with 95% confidence intervals (CIs) calculated for each demographic subgroup. Age- and sex-specific percentile curves were subsequently generated. A multivariable linear regression model was employed to examine the associations between PhA and potential determinants, including age, sex, body mass index (BMI), and other clinically relevant covariates.

**Results:**

A total of 1,247 children were included in this study, with 689 (55.3%) being boys. No significant differences in PhA were detected between boys and girls in the 6- and 7-year-old age groups. However, boys consistently exhibited higher PhA values than girls in the 8–11-year-old age groups (*p* < 0.05). PhA increased with age in both sexes, with similar age-related trends and percentile curves. The regression model revealed that age (B = 0.018, 95%CI: 0.003, 0.033) and BMI (B = 0.077, 95%CI: 0.068, 0.086) were positively correlated with PhA, whereas extracellular water (ECW) / intracellular water (ICW) (B = −22.925, 95%CI: −25.150, −20.699) had a negative effect on PhA.

**Conclusion:**

This study delineates the characteristics of PhA in Chinese children aged 6 to 11 years. The newly established reference ranges offer clinicians and researchers a practical tool for evaluating PhA in this pediatric population. These findings provide a valuable foundation for clinical assessment and further research on PhA in children.

## 1 Introduction

Bioelectrical impedance analysis (BIA) is a non-invasive and convenient technique for measuring human body composition and has gained widespread application. It is utilized to analyze body composition, assess nutritional status, and evaluate and monitor the effects of nutritional treatments (1–4).

**Figure 1:**
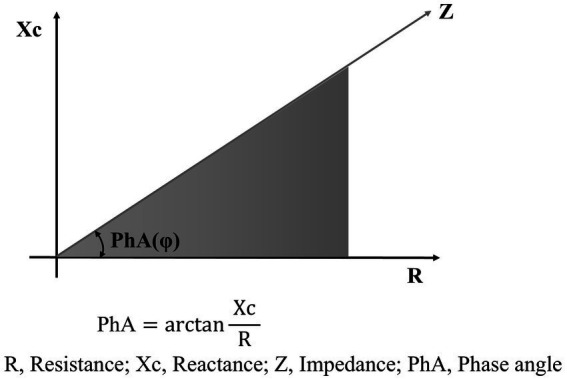
Schematic diagram of phase angle.

BIA measures resistance (R) and reactance (Xc) using a weak alternating current as it passes through body tissues (5). R reflects the opposition to the flow of electrical current and is inversely related to the water and electrolyte content of the tissues. Xc represents the capacitive effect of cell membranes. Body composition can be indirectly estimated using prediction equations derived from R and Xc measurements. Since bioelectric impedance (Z) is the vector sum of R and Xc, phase angle (PhA) is defined as the angle between Z and R (Figure 1) (5).

PhA has become a valuable independent indicator of nutritional status, characterized by its high sensitivity and specificity (6, 7). Accumulating evidence demonstrates that PhA is closely associated with total hospital length of stay (LOS) and hospitalization costs (8–10). Moreover, PhA has been shown to be a reliable predictor of clinical outcomes, including mortality and morbidity, across various patient populations (11–15). These findings underscore the importance of PhA as a non-invasive tool for nutritional assessment and clinical decision-making.

Due to the influence of multiple factors, including age, sex, ethnicity, and developmental status, there is currently no established normal range for PhA in children. Studies involving PhA in pediatric populations generally utilize normal control groups to analyze the status of PhA under disease conditions and to assess therapeutic effects (16, 17).

Exploring the range of PhA in school-aged children in China and analyzing its relationship with body mass index (BMI), age, and sex are essential. This study aimed to explore the potential application of PhA and to provide clinical professionals with accurate parameters for assessing nutritional status and guiding precision nutrition therapy in school-aged children.

## 2 Materials and methods

### 2.1 Study design and population

A retrospective cross-sectional study was conducted involving children who underwent body composition analysis via BIA at the Department of Health Care Center from June 2021 to December 2023.

The inclusion criteria were as follows: age between 6 and 11 years, absence of nutritional diseases, and completion of a BIA test.

The exclusion criteria included children who were underweight, overweight, or obese; those with extreme height (either very short or excessively tall); and those with fever, infectious diseases, or known chronic conditions (e.g., tuberculosis, hepatic or renal insufficiency, complex congenital heart disease, diabetes mellitus, anemia, or immunopathies). Known chronic diseases were identified from the past medical history section of outpatient records in the hospital information system (HIS).

This study received approval from the Ethics Committee of Beijing Children’s Hospital, Capital Medical University (2024-E-198-R), with a waiver of informed consent.

### 2.2 Methods

#### 2.2.1 Data collection and quality control

This study used the unique identification code from the hospital’s medical information system (identity document, ID), and the patient’s registration card number served as the ID for body composition analysis. This approach replaced the system-generated sequential test codes with real-name characteristic codes to ensure that the test data strictly corresponded to individual identities. Clinical data and laboratory results were obtained from the HIS of the outpatient department and were verified through a dual-check process conducted by different medical workers.

The window for laboratory tests was set at 2 weeks before and after the BIA measurement. When there were more than one test results, the one closest to the BIA measurement was selected.

Body composition data were measured using a bioelectrical impedance analyzer (InBody 770, Biospace, Korea) (18). Height was measured in a standing position with an accuracy of 0.1 cm, while weight was measured using the BIA device itself, with an accuracy of 0.1 kg. In general, the BIA test requires fasting or limiting food and water intake for more than 2 h, emptying the bowels and bladder as much as possible, and wearing lightweight underwear.

Finally, the following parameters were obtained: body fat mass (BFM), lean body mass (LBM) or fat-free mass (FFM), skeletal muscle mass (SSM), intracellular water (ICW), extracellular water (ECW), percentage body fat (PBF), visceral fat area (VFA), and 50 kHz whole-body PhA. BMI was calculated as W (kg) /H^2^ (m), while FFMI was calculated as FFM (kg) /H^2^ (m).

#### 2.2.2 Assessment of height and nutrition status

The assessment of height was based on the growth curves of children from nine provinces and cities in China (2005) (19). Children with heights above the 97th percentile for their age and sex were classified as tall, while those below the 3rd percentile were classified as short. Heights within the 3rd to 97th percentile (P3–P97) range were considered normal.

**Figure 2:**
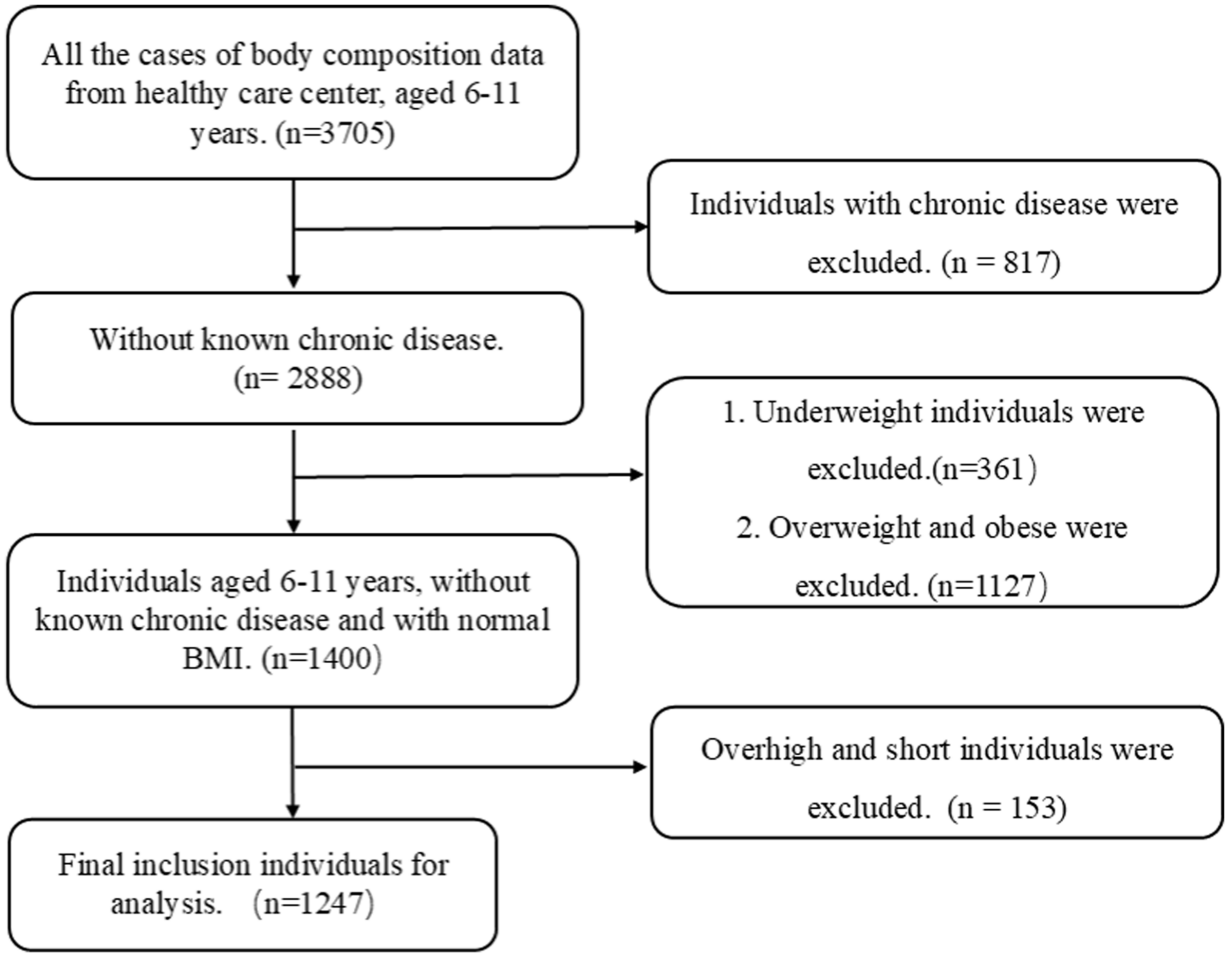
Flowchart depicting participant selection. BMI, body mass index.

Nutritional status was assessed according to the current Chinese health industry standards: “Screening for Overweight and Obesity among School-age Children and Adolescents” (20) and “Screening Standard for Malnutrition of School-age Children and Adolescents” (21). These standards were used to classify the children as underweight (moderate and mild underweight), normal weight, or overweight/obesity (overweight and obesity). The workflow chart is shown in Figure 2.

#### 2.2.3 Statistical analysis

Continuous variables were expressed as mean ± standard deviation (SD). One-way ANOVA with least significant difference (LSD) *post hoc* testing was performed for multiple group comparisons, applied when ANOVA showed significant differences at *p* < 0.05. An independent samples *t*-test was performed for comparisons between two groups. All these analyses were conducted using SPSS Statistics (version 25.0) (SPSS, Inc., Chicago, IL, USA), with a two-tailed *p*-value < 0.05 considered statistically significant.

Age- and sex-specific percentile curves of PhA were generated using the generalized additive model for location, scale, and shape (GAMLSS) model, employing either the Box-Cox power exponential (BCPE) or Box-Cox t (BCT) distribution, combined with cubic spline smoothing. Both distributions have four parameters: *μ*, *σ*, *ν*, and *τ*, representing location (median), scale (approximate coefficient of variation), skewness (power transformation for symmetry), and kurtosis (degrees of freedom or power exponent), respectively. The model fit was assessed using the Bayesian information criterion and Q-Q plots. The final best-fitting model for PhA in both boys and girls was based on the BCT distribution. Data analyses were performed using the R package “GAMLSS” in R 4.4.2.

The influencing factors were selected as independent variables, and the PhA of children was used as dependent variables. The multiple linear regression model was used for data analysis (22). The effect size, Cohen’s ƒ^2^ = R^2^/(1-R^2^), was calculated to evaluate the strength of the association between the predictor and the children’s PhA, where 0.02 ≤ ƒ^2^ < 0.15 indicated a small effect size, 0.15 ≤ ƒ^2^ < 0.35 indicated a medium effect size, and ƒ^2^ ≥ 0.35 indicated a large effect size (23). The bilateral test level was *α* = 0.05.

## 3 Results

### 3.1 Sample characteristics

A total of 1,247 children were included in this study, comprising 689 boys (55.3%) and 558 girls (44.7%). Hemoglobin, albumin, aspartate transaminase, blood urea nitrogen, uric acid, and vitamin D levels were all within normal ranges across all age groups, and there was no statistically significant difference (*p* > 0.05). Detailed information is provided in Table 1. The parameters of body composition are shown in Table 2.

**Table 1:** Laboratory tests for each age group.

Items	6 years	7 years	8 years	9 years	10 years	11 years	Total
N (Male)	288 (170)	211 (118)	211 (110)	206 (114)	170 (94)	161 (83)	1,247 (558)
Hemoglobin (g/L)	129.5 ± 10.3	132.2 ± 9.3	132.3 ± 9.7	134.8 ± 8.1	134.6 ± 8.6	135.1 ± 8.7	132.6 ± 9.5
Total Protein (g/L)	68.5 ± 3.3	71.0 ± 4.7	72.1 ± 3.9	75.4 ± 5.2	73.2 ± 5.0	71.9 ± 5.2	71.9 ± 5.0
Albumin (g/L)	43.3 ± 3.0	44.3 ± 2.3	44.5 ± 2.4	44.6 ± 2.6	44.5 ± 1.9	44.4 ± 2.2	44.2 ± 2.5
Prealbumin (mg/L)	177.4 ± 32.5	188.1 ± 38.8	187.0 ± 26.2	197.2 ± 30.9	189.1 ± 31.7	199.3 ± 31.5	188.7 ± 32.6
AST (U/L)	30.2 ± 5.1	29.3 ± 5.1	29.6 ± 6.4	27.4 ± 4.8	31.5 ± 4.5	29.2 ± 4.8	29.4 ± 5.3
Urea Nitrogen (mmol/L)	4.6 ± 1.1	4.9 ± 1.0	4.6 ± 1.2	4.7 ± 0.8	4.4 ± 1.0	14.7 ± 6.3	5.7 ± 2.0
Uric Acid (umol/L)	259.1 ± 58.5	276.4 ± 54.5	284.1 ± 52.8	338.4 ± 94.8	300.1 ± 94.5	318.0 ± 71.0	293.7 ± 76.4
Vitamin D (mmol/L)	59.0 ± 15.7	61.8 ± 17.1	59.9 ± 17.0	57.9 ± 13.4	53.1 ± 16.0	52.3 ± 16.5	57.5 ± 16.3

AST, Aspartate Aminotransferase.

**Table 2:** Characteristics of human body composition.

Items	Male						Female					
	6 years (*n* = 170)	7 years (*n* = 118)	8 years (*n* = 110)	9 years (*n* = 114)	10 years (*n* = 94)	11 years (*n* = 83)	6 years (*n* = 118)	7 years (*n* = 93)	8 years (*n* = 101)	9 years (*n* = 92)	10 years (*n* = 76)	11 years (*n* = 78)
Height (cm)	118.90 ± 4.77	123.73 ± 4.66	129.45 ± 5.24	138.2 ± 6.50	141.83 ± 5.46	144.97 ± 5.96	117.70 ± 5.45	123.59 ± 5.62	130.62 ± 5.43	136.39 ± 8.41	143.49 ± 6.28	149.58 ± 6.12
BMI (kg/m^2^)	15.08 ± 1.65	15.56 ± 2.41	16.55 ± 2.87	18.46 ± 3.92	17.83 ± 3.24	17.88 ± 3.07	14.97 ± 1.68	15.18 ± 1.82	16.35 ± 2.58	17.18 ± 2.52	17.00 ± 2.13	18.04 ± 3.18
TBW (kg)	13.30 ± 1.39	14.54 ± 1.75	16.39 ± 2.24	19.50 ± 3.07	20.44 ± 2.87	21.51 ± 3.13	12.48 ± 1.49	13.97 ± 1.66	15.90 ± 1.86	18.10 ± 2.91	20.13 ± 2.58	22.56 ± 3.53
BFM (kg)	3.41 ± 2.12	4.20 ± 3.17	5.73 ± 4.42	9.19 ± 6.49	8.38 ± 5.46	8.53 ± 5.35	3.60 ± 2.09	4.27 ± 2.54	6.35 ± 3.63	7.51 ± 3.82	7.68 ± 3.13	9.95 ± 5.49
FFM (kg)	18.05 ± 1.89	19.77 ± 2.36	22.29 ± 3.04	26.57 ± 4.18	27.84 ± 3.90	29.31 ± 4.24	16.98 ± 2.02	19.04 ± 2.25	21.68 ± 2.51	24.67 ± 3.98	27.42 ± 3.47	30.73 ± 4.81
SMM (kg)	8.63 ± 1.13	9.65 ± 1.42	11.15 ± 1.82	13.62 ± 2.46	14.37 ± 2.28	15.25 ± 2.55	7.99 ± 1.20	9.20 ± 1.34	10.74 ± 1.52	12.51 ± 2.36	14.08 ± 2.05	16.02 ± 2.84
FFMI (kg/m^2^)	11.17 ± 4.03	9.83 ± 5.17	10.49 ± 4.97	9.20 ± 5.81	11.82 ± 4.71	11.51 ± 4.79	10.64 ± 3.95	10.20 ± 4.44	10.64 ± 4.39	11.39 ± 4.24	11.81 ± 4.08	12.78 ± 3.40
BFP (%)	15.17 ± 5.85	16.25 ± 7.00	16.78 ± 6.96	23.13 ± 9.89	21.34 ± 8.76	21.17 ± 8.28	17.78 ± 6.07	17.45 ± 6.90	18.67 ± 8.13	22.39 ± 7.15	21.30 ± 6.38	23.22 ± 7.04
VFA (cm^2^)	16.92 ± 11.63	14.98 ± 13.80	17.83 ± 18.55	21.73 ± 25.96	31.97 ± 30.13	26.24 ± 20.38	16.22 ± 9.66	15.35 ± 8.55	22.68 ± 16.25	26.29 ± 19.42	29.75 ± 16.06	41.92 ± 29.27
BMC (kg)	4.07 ± 6.46	6.53 ± 8.49	6.64 ± 9.06	10.18 ± 11.32	5.59 ± 8.02	6.74 ± 9.62	3.99 ± 6.56	5.80 ± 8.15	5.87 ± 8.18	5.19 ± 8.13	5.28 ± 7.54	4.41 ± 6.83

BMI, body mass index; TBW, total body water; BFM, body fat mass; FFM, fat-free mass; SMM, skeletal muscle Mass; FFMI, Fat-free mass index; PBF, percentage Body fat; VFA, visceral fat area; BMC, bone mass content.

### 3.2 Distribution of PhA and age- and sex-specific percentiles

**Figure 3:**
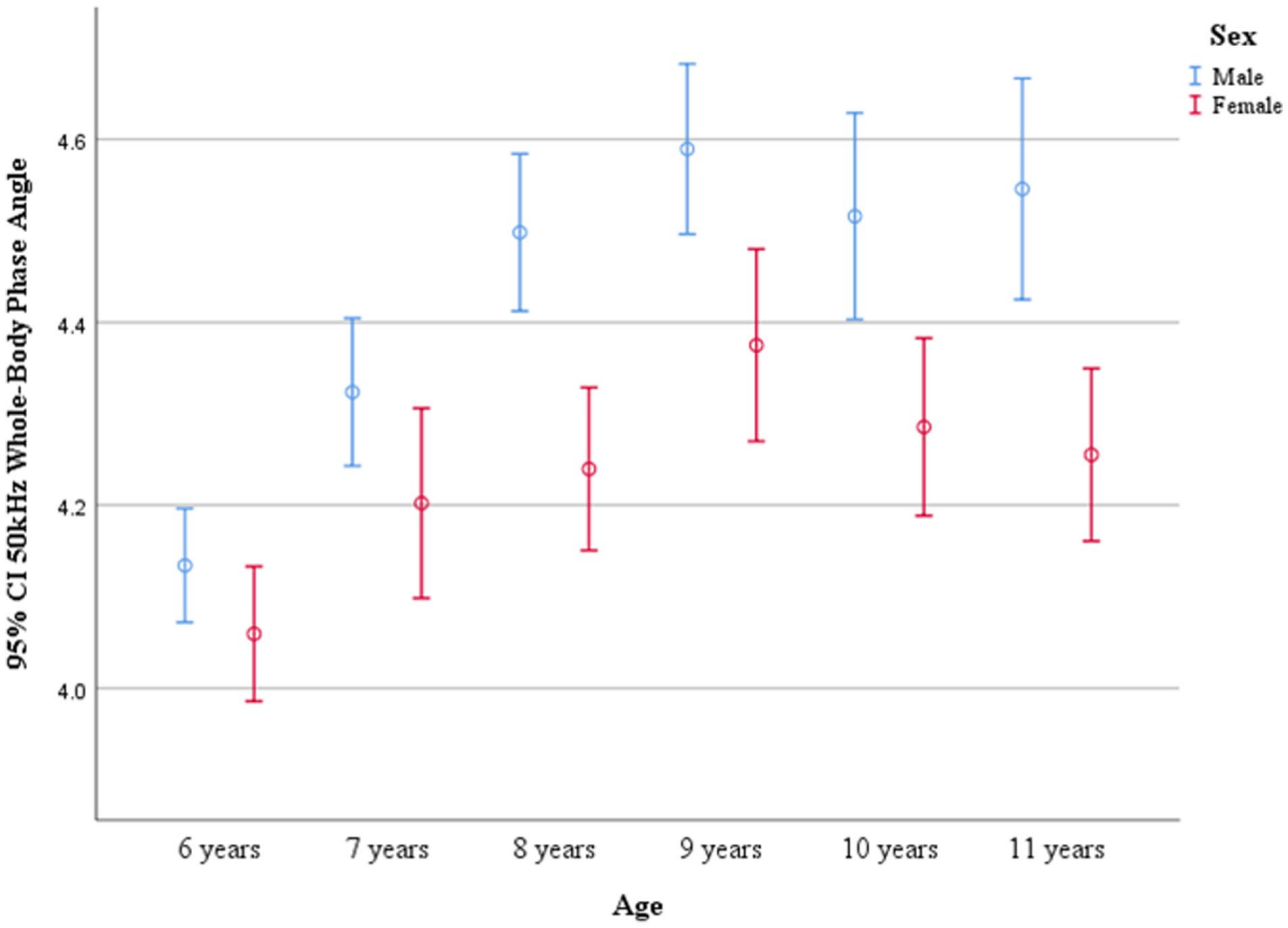
95% CIs of whole-body phase angle at 50 kHz across age groups.

**Figure 4:**
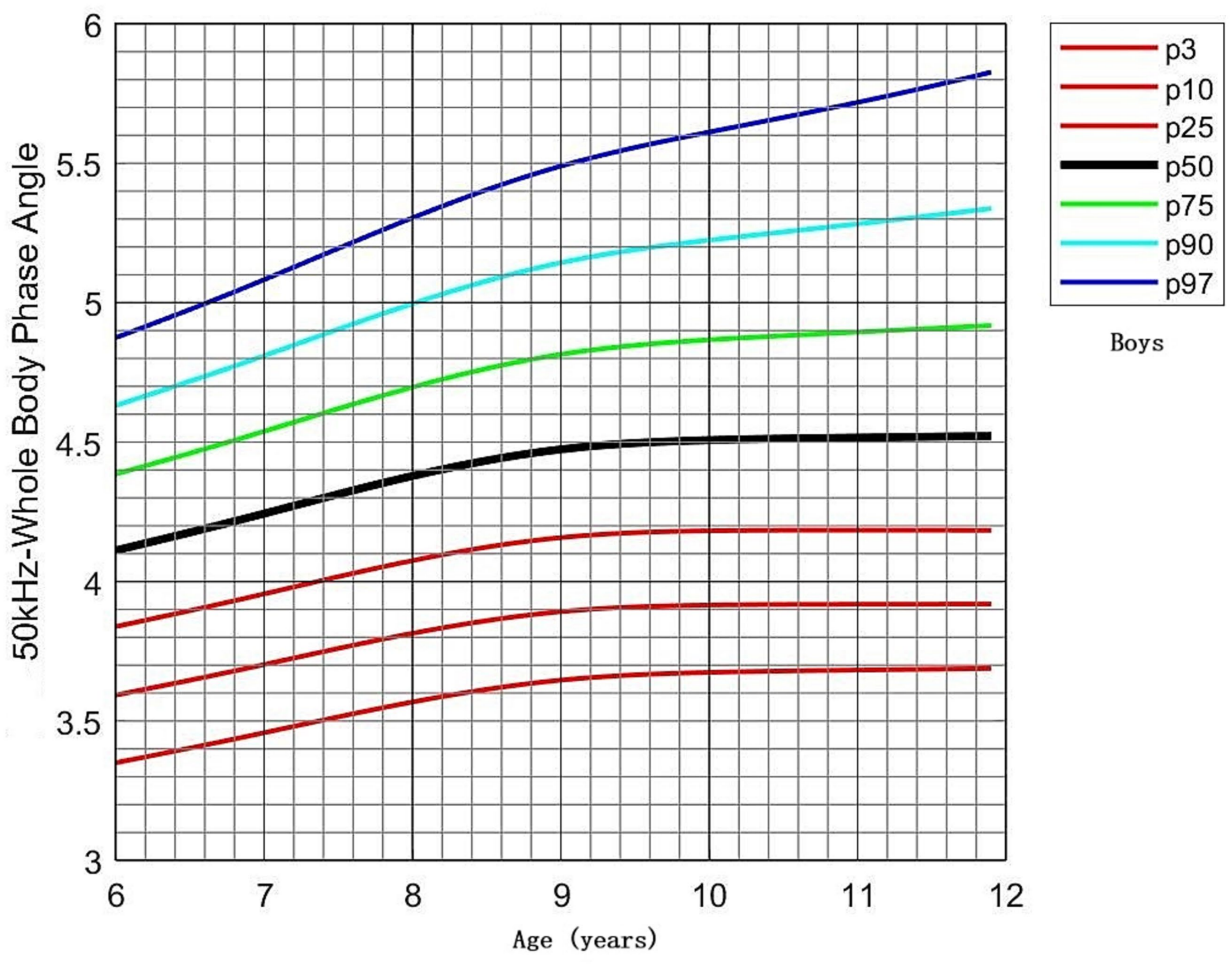
Percentile curves of whole-body phase angle at 50 kHz for the Chinese male children aged 6–11 years.

**Figure 5:**
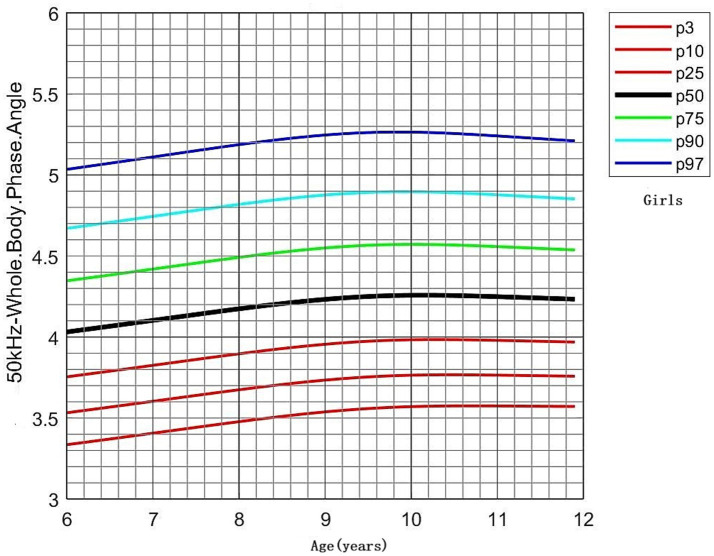
Percentile curves of whole-body phase angle at 50 kHz for the Chinese female children aged 6–11 years.

PhA values by age and sex are shown in Table 3. No significant differences in PhA were observed between boys and girls in the 6- and 7-year-old groups. However, boys consistently exhibited higher PhA values than girls in the 8–11-year-old groups (*p* < 0.05). PhA increased with age, as evidenced by the results of one-way ANOVA (*p* < 0.001). In boys, PhA levels in the 8–11-year-old group were significantly higher than those in the 6- and 7-year-old groups (*p* < 0.05). In girls, PhA values were higher in all age groups from 7 to 11 years compared to the 6-year-old group (*p* < 0.05). Distribution plots of PhA with 95% confidence intervals (CIs) by age and sex are presented in Figure 3. The smoothed age-specific centile curves and values for PhA among boys and girls are illustrated in Figures 4, 5, with some details provided in Supplementary Table 1.

**Table 3:** Whole-body phase angle at 50 kHz across age groups.

Age	Male	Female	*t-test*	*P -*value
6 years	4.13 ± 0.41	4.06 ± 0.40	−1.530	0.127
7 years	4.32 ± 0.44^a^	4.20 ± 0.50 ^a^	−1.861	0.064
8 years	4.50 ± 0.46^a b^	4.24 ± 0.45 ^a^	−4.139	0.000
9 years	4.59 ± 0.50^a b^	4.38 ± 0.51^a b c^	−3.035	0.003
10 years	4.52 ± 0.55^a b^	4.29 ± 0.43^a^	−3.076	0.002
11 years	4.55 ± 0.55^a b^	4.26 ± 0.42^a^	−3.771	0.000
*F* ^#^	18.296	5.578		
*P -*value	0.000	0.000		

F = F-statistic. One-way ANOVA was used to assess the differences in phase angle values among the age groups. If there was a statistical difference (*P* < 0.05), the LSD method was applied for pairwise comparisons. The superscript “a, b, c, d, e” respectively indicate that the current age group was compared with the 6-year-old group, 7-year-old group, 8-year-old group, 9-year-old group, 10-year-old group within the same gender, with observed statistical significance (*P* < 0.05).

### 3.3 Establishment of a multiple linear regression equation

Model 1: A regression model was established to predict 50 kHz whole-body PhA using age, sex, and BMI: 50 kHz whole-body PhA = 2.97 + 0.02 × Age + 0.08 × BMI – 0.15 × Sex (Male = 0, Female = 1).

The model showed a significant predictive effect (R = 0.509, R^2^ = 0.259, *F* = 144.7, *p* < 0.001, ƒ^2^ = 0.35), indicating that age, sex, and BMI collectively have substantial explanatory power for PhA. The model revealed that age (B = 0.018, 95%CI: 0.003, 0.033) and BMI (B = 0.077, 95%CI: 0.068, 0.086) were positively correlated with PhA.

Model 2: Based on the principles of PhA and its clinical influencing factors, a comprehensive linear regression model was developed by identifying potential parameters associated with PhA and optimizing the model indicators: 50 kHz whole-body PhA = 17.56 + 0.039 × Age + 0.84 × BMI – 0.073 × Sex (Male = 0, Female = 1) – 22.925 × (ECW/ICW) – 0.009 × VFA.

This model demonstrated a strong predictive capacity (R = 0.879, R^2^ = 0.773, *F* = 200.571, *p* < 0.001, ƒ^2^ = 3.41), indicating that the inclusion of multiple parameters significantly enhances the explanatory power for PhA (Table 4). The model revealed that ECW/ICW (B = −22.925, 95%CI: −25.150, −20.699) and VFA (B = −0.009, 95%CI: −0.010, −0.007) had negative effects on PhA.

**Table 4:** Multiple linear regression analysis of age, sex, BMI, and body composition parameters with PhA in children aged 6–11 years.

Variables	Model 1				Model 2			
	B 95%CI	β	*t*	*P*-value	B 95%CI	β	*t*	*P*-value
Constant	2.972 (2.818,3.127)		37.716	0.000	17.562 (15.966, 19.158)		21.642	0.000
Age	0.018 (0.003,0.033)	0.061	2.294	0.022	0.039 (0.020, 0.057)	0.114	4.116	0.000
Sex^*^	−0.152 (−0.199, −0.104)	−0.152	−6.214	0.000	−0.073 (−0.129, −0.017)	−0.066	−2.550	0.011
BMI	0.077 (0.068, 0.086)	0.451	16.963	0.000	0.084 (0.075, 0.093)	0.589	18.581	0.000
ECW/ICW					−22.925 (−25.150, −20.699)	−0.536	−20.699	0.000
VFA					−0.009 (−0.010, −0.007)	−0.430	−14.945	0.000
Alb					−0.006 (−0.017, 0.005)	−0.027	−1.038	0.300

* Male: 0, Female:1. Male children are used as the reference group. BMI, body mass index; PhA, Whole-body Phase Angle at 50 kHz; ECW, extracellular water; ICW, intracellular water; VFA, visceral fat area; ALB, albumin.

## 4 Discussion

The present study provides valuable insights into the distribution of PhA in Chinese children aged 6 to 11 years and establishes a reference range for PhA in this pediatric population. Age is a significant factor influencing PhA values. In addition, sex differences in PhA were consistent across all age groups, with boys consistently exhibiting higher PhA values than girls.

PhA in children and adolescents is influenced by age, sex, and ethnicity. In the MoMo study conducted by S.C.E. Schmidt et al. (24), which focused on German children aged 6 to 10 years, PhA values were reported as 5.46° (SD = 0.46) for boys and 5.34 ° (SD = 0.40) for girls. In contrast, our study on Chinese children aged 6 to 11 years observed lower PhA values compared to those reported by De Palo et al. (25) in Italy, who found PhA values of 5.5°(SD = 0.6) for 6- to 7-year-old children, 5.7(SD = 0.6)° for 8-year-old children, 5.7(SD = 0.6) °for 9-year-old children, and 5.8 (SD = 0.5) ° for 10- to 11-year-old children. In addition, Nescolarde et al. (26) reported PhA values of 5.7° (SD = 0.4)for 6- to 7-year-olds, 5.8° (SD = 0.6)for 8- to 9-year-olds, and 5.9 ° (SD = 0.5)for 10- to 11-year-olds. These values were higher than those reported in our Chinese cohort, suggesting that ethnicity may be a significant factor influencing differences in PhA values.

The consistent pattern observed in boys having higher PhA values than girls was also observed in these studies. Although the MoMo study (24) did not provide specific PhA values for each age group, the overall trend in PhA changes was similar to our findings—showing an increase from ages 6 to 8 years, followed by a plateau from ages 9 to 11 years.

PhA is also influenced by the degree of pubertal development. The level of hormones can affect cell status and water distribution. It can also influence the distribution of fat mass. In this study, although some girls had begun the process of puberty, the stages of development were not classified. De Moraes et al. investigated the relationship between pubertal development and PhA changes in children aged 10 to 15 years, finding that PhA increased with the progression of puberty, consistent with the pattern that boys have higher PhA values than girls (27).

The clinical application of PhA is widespread, with some studies focusing on patients with chronic diseases and those in intensive care units (ICUs) (11, 12, 28). Sonoko et al. (12) conducted a retrospective analysis of 501 patients who underwent surgical treatment for gastrointestinal, hepatobiliary, and pancreatic malignancies. The patients were stratified into four groups based on PhA quartiles: high PhA (>75th percentile, Q4), low PhA (≤25th percentile, Q1), and normal PhA (≤75th and >25th percentiles, Q2 and Q3). Multivariate regression analysis revealed that low PhA was an independent risk factor for severe complications, ICUs length of stay, and mortality. In pediatric intensive care units (PICUs), PhA has been utilized to predict mortality outcomes. Zamberlan et al. (11) and Yang et al. (28) identified PhA cut-off values of 2.8° and 3.1°, respectively, for predicting mortality in critically ill children. Both studies demonstrated that lower PhA values were associated with significantly higher mortality rates, and Cox regression analysis confirmed PhA as an independent risk factor for mortality in these patients (11, 28). Furthermore, PhA has been identified as an independent prognostic factor in pediatric patients with juvenile idiopathic arthritis (16) and inflammatory bowel disease (17), highlighting its close association with clinical outcomes.

Although some studies on PhA have been conducted in pediatric subspecialties, standardized reference ranges based on age and sex have not yet been established. In clinical practice, normal control groups are often required for comparison (16, 17). Some studies have employed age- and sex-matched controls, which has added to the complexity of the research. In this study, children with normal BMI were selected, and PhA was described separately according to age and sex. This study preliminarily established a normal range for PhA in Chinese children. As such, it provides a valuable reference for future PhA studies in Chinese pediatric populations and offers clinicians an additional nutritional indicator for clinical practice. This research further promotes the broader application of PhA in clinical settings.

In addition to clarifying the influencing factors of PhA, it is also necessary to define the considerations for its clinical application. This study explored the relationship between PhA and age, sex, BMI, and selected body composition parameters. Although all participants had normal BMI values, BMI showed the strongest relationship with PhA, while age showed a positive correlation. In addition, sex differences were observed, with female children having lower PhA values than the male children. In addition to anthropometric measurements, the study further analyzed the interrelationships betweenvarious body composition parameters and established regression equations. The model indicated that PhA may have significant limitations in cases of disrupted homeostasis between ECW and ICW, such as edema and ascites (29, 30).

## 5 Conclusion

These results establish age-specific reference values for PhA in children with normal BMI and height, offering a clinically useful benchmark for pediatric health assessments. The findings provide an evidence-based foundation for clinicians to interpret PhA measurements in this population, facilitating more accurate evaluations of nutritional status and cellular health in children.

### 5.1 Limitations

This was a retrospective study. Although BIA data collection recommends fasting for more than 2 h, there may have been biases during the measurement. This study involved only healthy children from a single center and therefore cannot fully represent the entire population, including both urban and rural areas in China. It merely provides a preliminary description of the PhA status of healthy children in China.

This study focused solely on school-aged children. Although it included children in early puberty, it did not assess their pubertal development stages or explore the relationship between pubertal status and PhA. In addition, detailed characterization of PhA in preschool-aged children and those with non-normal BMI values is needed in clinical practice.

## Data Availability

The original contributions presented in the study are included in the article/Supplementary material, further inquiries can be directed to the corresponding author.
