# Supplementary material for: Reference percentiles for evaluating phase angle using bioelectrical impedance analysis in Chinese children aged 6–11 years
Source: Front Nutr. 2025 Jul 31;12:1597087. doi: 10.3389/fnut.2025.1597087 (PMC12350123; doi:10.3389/fnut.2025.1597087)
Supplement: Supplementary file 1 [file Table_1.pdf]

Supplementary Table 1. Percentile table of whole-body phase angle at 50 kHz (°)

Age	Gender	P2.5	P5	P10	P25	P27	P33	P50	P67	P73	P75	P90	P95	P97.5
6years	Male	3.33	3.50	3.60	3.90	3.90	4.00	4.15	4.30	4.40	4.40	4.60	4.80	5.00
	Female	3.30	3.40	3.59	3.80	3.80	3.90	4.00	4.27	4.30	4.40	4.60	4.70	5.10
7years	Male	3.50	3.60	3.79	4.00	4.01	4.20	4.30	4.50	4.60	4.60	4.90	5.01	5.21
	Female	3.24	3.47	3.60	3.80	3.90	4.00	4.20	4.40	4.46	4.50	5.06	5.10	5.10
8years	Male	3.68	3.80	3.91	4.20	4.20	4.30	4.50	4.70	4.80	4.80	5.10	5.35	5.60
	Female	3.60	3.60	3.70	3.90	3.95	4.00	4.10	4.40	4.45	4.50	4.90	5.19	5.39
9years	Male	3.69	3.80	4.00	4.28	4.30	4.40	4.50	4.80	4.90	4.90	5.30	5.53	5.63
	Female	3.53	3.60	3.80	4.00	4.00	4.10	4.30	4.53	4.60	4.70	5.17	5.34	5.50
10years	Male	3.44	3.58	3.90	4.28	4.30	4.30	4.40	4.70	4.80	4.90	5.15	5.65	5.90
	Female	3.39	3.595	3.77	4.00	4.00	4.10	4.25	4.40	4.50	4.50	4.80	5.02	5.25
11years	Male	3.70	3.72	3.90	4.20	4.20	4.20	4.40	4.70	4.80	4.90	5.30	5.68	6.06
	Female	3.59	3.60	3.70	4.00	4.10	4.10	4.20	4.30	4.47	4.50	4.80	5.01	5.31
